# Hiding Lipid Presentation: Viral Interference with CD1d-Restricted Invariant Natural Killer T (iNKT) Cell Activation

**DOI:** 10.3390/v4102379

**Published:** 2012-10-23

**Authors:** Daniëlle Horst, Ruben J. Geerdink, Anna M. Gram, Arie J. Stoppelenburg, Maaike E. Ressing

**Affiliations:** 1 Department of Medical Microbiology, University Medical Center Utrecht, 3584 CX Utrecht, The Netherlands; Email: d.horst-2@umcutrecht.nl (D.H.); r.j.geerdink@students.uu.nl (R.J.G.); a.m.gram@umcutrecht.nl; 2 Department of Pediatric Immunology, University Medical Center Utrecht, 3584 CX Utrecht, The Netherlands; Email: A.J.Stoppelenburg@umcutrecht.nl (A.J.S.)

**Keywords:** CD1d, iNKT cells, immune evasion, viruses, antigen presentation

## Abstract

The immune system plays a major role in protecting the host against viral infection. Rapid initial protection is conveyed by innate immune cells, while adaptive immunity (including T lymphocytes) requires several days to develop, yet provides high specificity and long-lasting memory. Invariant natural killer T (iNKT) cells are an unusual subset of T lymphocytes, expressing a semi-invariant T cell receptor together with markers of the innate NK cell lineage. Activated iNKT cells can exert direct cytolysis and can rapidly release a variety of immune-polarizing cytokines, thereby regulating the ensuing adaptive immune response. iNKT cells recognize lipids in the context of the antigen-presenting molecule CD1d. Intriguingly, CD1d-restricted iNKT cells appear to play a critical role in anti-viral defense: increased susceptibility to disseminated viral infections is observed both in patients with iNKT cell deficiency as well as in CD1d- and iNKT cell-deficient mice. Moreover, viruses have recently been found to use sophisticated strategies to withstand iNKT cell-mediated elimination. This review focuses on CD1d-restricted lipid presentation and the strategies viruses deploy to subvert this pathway.

## 1. Introduction

Viruses rely on host cells for their replication. They manipulate infected cells to optimize the production of infectious virions. At the same time, the synthesis of viral proteins renders infected cells vulnerable to detection and elimination by the host’s immune system. To thwart immune eradication, viruses are equipped with various immune evasive functions. Many of the immune evasion strategies discovered so far interfere with T cell recognition of virus-producing cells [1,2,3,4]. This reflects the prominent role of T cell responses in the control of viral infection. Activation of virus-specific T cells depends on the detection of viral peptides presented by MHC class I or class II molecules. 

**Figure 1:**
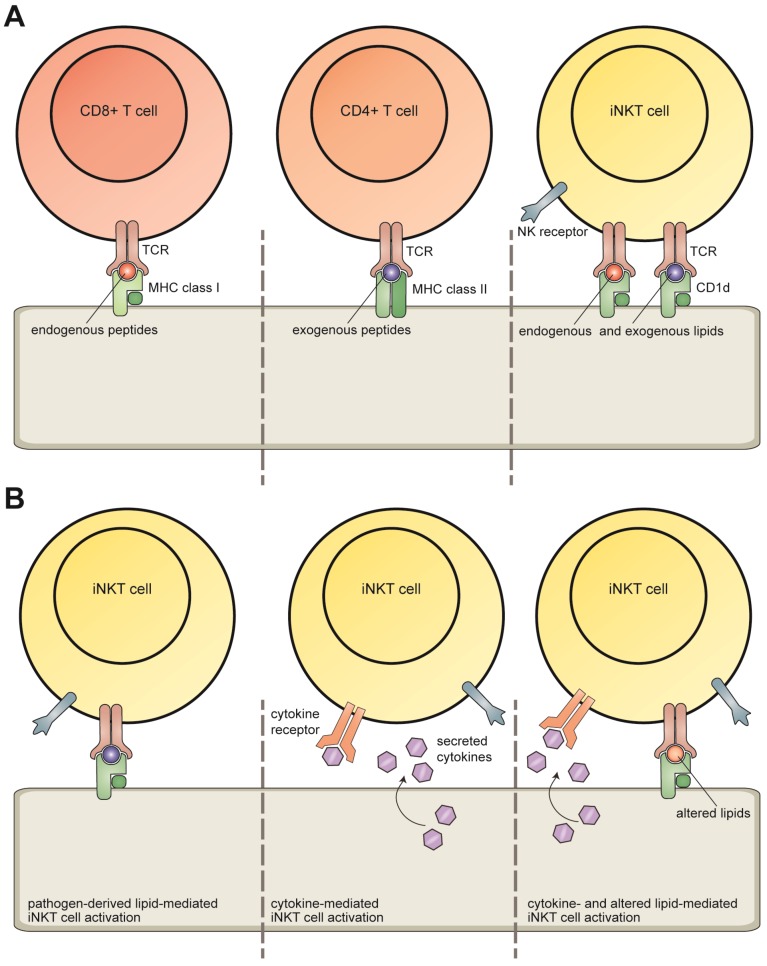
(**A**) MHC- and CD1d-restricted antigen presentation. In general, MHC class I molecules present endogenous peptides to CD8^+^ T cells, whereas MHC class II molecules present exogenous peptides to CD4^+^ T cells. Both endogenous and exogenous lipids are presented by the non-classical MHC molecule CD1d to invariant natural killer T (iNKT) cells. (**B**) iNKT cell activation. iNKT cells can be activated by pathogen-derived lipid antigens, innate or cytokine signals, and a combination of altered self-lipid antigens and cytokine signals.

In addition to classical MHC molecules, non-classical MHC molecules exist, such as CD1 molecules (Figure 1A). The human CD1 family consists of five members divided into three groups: CD1a, CD1b, and CD1c form group 1, group 2 consists of CD1d, and group 3 encompasses CD1e. These CD1 isoforms fulfill distinct functions, as indicated by differences in intracellular trafficking pathways and antigen-binding specificities. CD1a-c and CD1d present lipid antigens to T cells, including natural killer T (NKT) cells and γδ T cells [5,6,7], whereas CD1e has been suggested to assist in lipid loading of the antigen-presenting CD1 molecules [8]. In mice, group 1 and 3 CD1 isoforms are absent and, therefore, mouse studies rely on CD1d function to model all CD1 isoforms. Murine group 2 CD1 includes two variants, CD1d1 and CD1d2, yet, most effector functions are CD1d1-dependent, also supported by the inability of CD1d2 to support NKT cell selection [9]. NKT cells recognize lipids presented by CD1d molecules and are divided into two subsets; invariant (type I) NKT (iNKT) cells and diverse (type II) NKT cells [7,10]. In this review, we focus on iNKT cells. These cells are of particular interest as their response bridges the innate and adaptive immune system by rapid secretion of vast amounts of polarizing cytokines. In recent years, evidence has been accumulating that iNKT cells contribute significantly to anti-viral defense. This role and the immune evasion strategies employed by viruses to subvert CD1d-restricted antigen presentation are the subject of this review. 

## 2. Invariant NKT Cells

iNKT cells form an important subpopulation of CD1d-restricted T cells. The name iNKT cell originates from concurrent cellular expression of receptors that hallmark NK cells and a (semi-) invariant T cell receptor (TCR), which consists of an invariant α-chain (Vα24-Jα18 in humans, Vα14-Jα18 in mice) paired with one of a limited set of β chains (Vβ11 in humans, Vβ2, Vβ7, or Vβ8 in mice). 

iNKT cells are activated in multiple ways (Figure 1B). Firstly, TCR engagement of CD1d complexes presenting pathogen-derived lipid antigens can lead to iNKT cell activation. Secondly, innate signals and cytokines such as IL-18 can activate iNKT cells independent of TCR signaling. The receptors NKG2D [11] and TIM-1 [12] can activate iNKT cells both independently and as co-stimulatory signals in concert with TCR triggering. Lastly, CD1d-mediated presentation of altered self-lipids in combination with cytokine signals can effectively activate iNKT cells. The mechanism of activation and the local polarizing cytokine environment dictate the subsequent iNKT cell response. 

Upon activation, iNKT cells rapidly produce large amounts of cytokines, including IFN-γ that has direct anti-viral effects and boosts NK cell activation. In addition, iNKT cells can mediate cytolysis, as a consequence of granzyme B, perforin, TRAIL, and FasL expression. Thus, iNKT cells could directly eliminate pathogens and tumor cells, although the *in vivo* importance of iNKT cell-induced cytotoxicity in general remains to be assessed. It is, however, clear that iNKT cells can mediate direct immune defense in the course of microbial infection, as was shown in *Salmonella typhimurium* and *Mycobacterium tuberculosis* infection models in mice [13,14,15], and as will be discussed in more detail below. Through preferential secretion of T_H_1 or T_H_2 cytokines, iNKT cells skew CD4^+^ T cell responses and determine the quality of ensuing adaptive immunity.

## 3. CD1d Antigen Presentation

CD1d molecules are composed of a heavy chain and β_2_-microglobulin (β_2_m). This structural homology with classical MHC class I molecules is reminiscent of the function shared by CD1d and MHC class I proteins, i.e. presenting antigens [16]. However, the diverse nature of antigens presented by either molecule is reflected in their antigen-binding grooves: whereas the grooves of highly polymorphic MHC class I are well-suited for binding defined peptides, lipid tails fit snugly into the hydrophobic pockets of CD1d, exposing the more polar moieties for TCR recognition. Association of lipid antigens with the CD1d binding groove is mainly mediated by non-specific, hydrophobic Van der Waals interactions, which might explain why CD1d molecules are non-polymorphic. Here, we will discuss the antigen presentation pathway of CD1d molecules in detail. 

Before lipids can be inserted into the antigen-binding groove of CD1d, they must first be extracted from the hydrophobic lipid bilayer into aqueous solution, a process that is facilitated by lipid transfer proteins. Distinct lipid transfer proteins vary in their modes of action and lipid-binding specificities [17]. Thus, lipid transfer proteins may facilitate preferential binding of certain lipid species by CD1d, thereby conferring a level of antigen selectivity. 

In addition, the route of CD1d trafficking influences the lipid repertoire presented by CD1d molecules. After association of CD1d heavy chains with β_2_m in the endoplasmic reticulum (ER), CD1d molecules travel via the Golgi compartment to the plasma membrane. The majority of CD1d leaves the ER in association with β_2_m, yet this association is not an absolute requirement for ER exit. In fact, surface expressed murine CD1d heavy chains are still capable of eliciting an NKT cell response in the absence of β_2_m [18,19]. However, human cells expressing predominantly free CD1d heavy chains displayed a significantly reduced ability to activate iNKT cells, suggesting that CD1d/β_2_m complexes are the functional unit of lipid antigen presentation in humans [20]. Furthermore, association of CD1d heavy chains with β_2_m is required for resistance to lysosomal degradation [21]. In this way, β_2_m might influence the lipid repertoire presented by CD1d molecules. 

Similar to MHC class II, CD1d molecules survey endocytic compartments for the presence of antigens. Endosomal targeting signals in the cytoplasmic tail of the CD1d heavy chain regulate its trafficking. A threonine-based sequence targets the lipid-presenting molecules to the plasma membrane. Removal of this signal from the CD1d tail or mimicking phosphorylation of the threonine residue redirects CD1d molecules to endolysosomal compartments [20]. A tyrosine-based sorting motif (YXXZ; Y is tyrosine, X a random amino acid, and Z a bulky hydrophobic amino acid) is required for internalization of surface CD1d complexes [22]. This motif is recognized by adaptor protein (AP)-2, directing CD1d to early endosomes [23]. In the mouse, CD1d molecules subsequently associate with AP-3, allowing murine CD1d to gain access to late endosomes and lysosomes [24]. In humans, the cytoplasmic tail of CD1d lacks the consensus sequence required for association with AP-3 [25]. As a result, human CD1d mostly surveys early endocytic compartments. Still, a fraction of CD1d molecules (both human and mouse) gains access to the endolysosomal system via an alternative trafficking pathway, relying on binding of CD1d with invariant chains or MHC class II/invariant chain complexes. A dileucine motif in the cytoplasmic tail of the invariant chain directs associated CD1d and/or MHC class II molecules to the endolysosomal system, including the MHC class II-loading compartment (MIIC) [26,27,28]. Due to the restricted expression of MHC class II molecules, this alternative CD1d trafficking pathway is mostly constrained to professional APCs, such as dendritic cells (DCs), macrophages, and B cells. Finally, the threonine-based targeting signal mediates re-expression of CD1d at the cell surface, where the CD1d molecules present their lipid cargo for surveillance by iNKT cells.

## 4. Lipid Antigens Presented by CD1d Molecules

CD1d molecules present both pathogen-derived lipids and self-lipids. The first CD1d-restricted lipid antigen found to activate iNKT cells was α-galactosylceramide (α-GalCer), a glycosphingolipid compound derived from marine sponges [29]. Originally, this lipid was identified in a screen for compounds with anti-tumor activity [30]. For a long time, α-GalCer remained the only known CD1d-restricted antigen and it is still widely used in functional assays. The last decade has provided insights into the nature of other, biologically relevant, lipid antigens capable of activating iNKT cells. 

A large diversity of self-lipid species bound to human CD1d has been identified by elution studies. Among those were glycerophospholipids having a variety of polar head groups and containing either one, two, or four radyl chains. Furthermore, several sphingomyelins and glycosylated sphingolipids were also eluted from CD1d molecules [31]. Thus, in contrast to the limited selection of peptides displayed by a particular MHC class I or class II allelic product, CD1d molecules appear to present a broad array of lipids.

### 4.1. Direct Recognition: Pathogen-Derived Lipids

In recent years, several studies demonstrated that CD1d presents pathogen-derived lipids capable of activating iNKT cells. The CD1d antigens α-glucuronosylceramide and α-galacturonosylceramide from *Sphingomonas* bacteria [32,33,34], α-galactosyldiacylglycerol from *Borrelia burgdorferi* [35], and α-glucosyldiacylglycerol from *Streptococcus pneumonia* [36] all contain α-linked glycans, a feature shared with α-GalCer. Since humans cannot synthesize α-linked glycolipids, an α-linked glycan could represent a specific antigenic determinant of CD1d-presented, pathogen-derived lipids. Besides these, different lipid species like cholesteryl α-glucoside from *Helicobacter pylori* [37] are presented by CD1d molecules and can activate iNKT cells. Since these lipid antigens are specific to microbes, they are non-self to the host’s immune system, which minimizes the chance of auto-reactivity.

### 4.2. Modulation of Self-Lipid Presentation during Viral Infection

The identity of physiologically relevant, stimulatory lipid antigens presented by CD1d in the context of viral infection remains, at this time, incompletely understood. As opposed to the above-mentioned microbial CD1d lipid antigens, virus-specific lipids do not exist. Therefore, the iNKT cell stimulatory lipids presented by CD1d during viral infection must be of host cell origin. This poses an intrinsic risk of undesired self-reactivity. To avoid this, self-lipids presented by CD1d should only be stimulatory towards iNKT cells during conditions of cellular stress, such as infection or carcinogenesis. Very recently, the cellular lipid profile was found to be altered during hepatitis B virus (HBV) infection, leading to increased activation of NKT cells. Whereas diverse NKT cells were stimulated by HBV-induced lysophosphatidylethanolamine, different lipid(s) were responsible for the activation of iNKT cells, although their nature was not identified [38].

Alterations in CD1d lipid presentation induced by viral infection appear linked to activation of pattern-recognition receptors, such as Toll-like receptors (TLRs). For example, the endosomal TLRs (TLR3, 7, 8, and 9) are involved in detecting viruses through the recognition of nucleic acid structures as pathogen-associated molecular patterns (PAMPs) [39,40]. TLR engagement could effectuate changes in CD1d antigen presentation in various ways, as discussed below. 

First, increased synthesis of antigenic self-lipids affects CD1d-mediated lipid presentation. Stimulation of myeloid DCs with (human) TLR8 or (mouse) TLR9 ligands resulted in enhanced iNKT cell activation, which was dependent on both CD1d expression and cytokine secretion (IL-12 in humans, type I interferons in mice). Two observations strongly support the view that increased synthesis of glycosphingolipids upon TLR stimulation causes enhanced CD1d presentation to iNKT cells: i) chemical inhibition of glycosphingolipid synthesis reverted the added iNKT stimulatory capacity provided by TLR-ligand-treated DCs and ii) mRNA levels for enzymes involved in glycosphingolipid synthesis were enhanced in TLR-ligand-stimulated DCs [41,42]. Muindi *et al*. conducted a more detailed study on the effects of TLR engagement on the repertoire of glycosphingolipids presented by murine CD1d. Both total cellular glycosphingolipid content and the pool of CD1d-bound glycosphingolipids were markedly altered following TLR4 stimulation of DCs. Surprisingly, the relative abundance of glycosphingolipids associated with CD1d did not mirror that in the total cellular lipid pool, suggesting selective lipid loading of CD1d molecules [43]. Accumulation of the self-lipid β-glucopyranosylceramide occurred during bacterial infection or upon TLR4 stimulation, leading to iNKT cell activation in a CD1d-dependent manner [44]. Whether these results can be extrapolated to viral infection remains to be determined. Still, stimulation of various TLRs, among which were the virus-sensing TLR3, 7, and 9, altered the expression of transcripts favoring β-glucopyranosylceramide synthesis, indicating that β-glucopyranosylceramide-mediated iNKT cell activation could contribute to anti-viral defense [44]. 

Second, reduced degradation of antigenic self-lipids influences the lipid repertoire presented by CD1d molecules. The lysosome-resident enzyme α-galactosidase A (α-Gal-A), known to be involved in lipid metabolism, appears to affect the generation of self-antigens recognized by iNKT cells, at least in mice [45]. Both *in vitro* and *in vivo*, α-Gal-A-deficient DCs stimulated iNKT cells in a CD1d-dependent manner to a greater extent than wild-type DCs. Incubation of wild-type DCs with the TLR4 ligand LPS or the bacterium *Listeria monocytogenes* caused a temporary decrease in α-Gal-A activity. This effect was abrogated in DCs deficient for the TLR-adaptor protein MyD88, indicating that TLR signaling is required for the observed decrease in α-Gal-A enzymatic activity. Moreover, stimulation of DCs with TLR4 and TLR9 ligands resulted in accumulation of glycosphingolipids. Thus, the TLR-dependent inhibition of α-Gal-A activity would provide a mechanistic link between TLR-mediated pathogen recognition and the generation, and subsequent presentation, of antigenic self-lipids by CD1d [45]. 

Third, the trafficking route of CD1d molecules affects lipid presentation. A subset (5–10%) of CD1d molecules associates with MHC class II complexes [28]. In TLR-stimulated mature DCs, MHC class II molecules are dramatically relocalized from intracellular endosomal compartments to the cell surface. As a consequence, the MHC class II-associated pool of CD1d molecules would not encounter certain lipid species along the endolysosomal route. In line with this, the presentation of exogenous lipid antigens by CD1d molecules is reduced for mature DCs [46]. In further support, mice that lack the MHC class II-associated invariant chain exhibit defects in the localization of MHC class II molecules to the endolysosomal route [47]. In these same invariant chain-deficient mice, CD1d-mediated lipid presentation of endosome-derived model antigen α-GalGalCer is also much reduced [15]. 

Finally, CD1d upregulation improves lipid antigen presentation to iNKT cells. CD1d mRNA levels were found to be increased upon infection of DCs with herpes simplex virus type 1 (HSV-1) or human cytomegalovirus (HCMV). UV-inactivated viruses caused a similar, although slightly weaker effect, suggesting that viral PAMPs are mainly responsible for the higher CD1d mRNA expression. Indeed, stimulation of TLR7 also elevated CD1d mRNA expression levels. The increased CD1d mRNA levels were accompanied by enrichment of CD1d proteins at the cell surface and enhanced activation and proliferation of iNKT cells [48]. 

In conclusion, TLR-mediated recognition of viral infection leads to altered lipid presentation by CD1d molecules, thereby affecting the activation of iNKT cells. The exact identity of the lipids presented by CD1d at the surface of virus-infected cells awaits further elucidation.

## 5. iNKT Cells in Anti-Viral Defense

CD1d-restricted antigen presentation to iNKT cells plays an important role in anti-viral defense. This is exemplified best by the observation that both patients with iNKT cell deficiency as well as CD1d- and iNKT cell-deficient mice are more susceptible to various viral infections (reviewed in [49,50]). 

iNKT cell-deficient patients are found to be particularly vulnerable to infection by herpesviruses. Two patients suffering from severe, disseminated Varicella-Zoster virus (VZV) infection after vaccination with a live-attenuated strain were shown to be deficient for iNKT cells [51,52]. Furthermore, X-linked lymphoproliferative syndrome (XLP), which is characterized by a high susceptibility to Epstein-Barr virus (EBV) infection, is caused by mutation of the gene encoding either signaling lymphocyte activation molecule (SLAM)-associated protein (SAP) or X-linked inhibitor of apoptosis (XIAP). Both SAP- and XIAP-deficient patients are almost completely devoid of iNKT cells, suggesting an important role for this cell population in the control of EBV infection [53,54,55]. Interestingly, Huck *et al*. investigated the immunodeficiency of two sisters who suffered from uncontrolled EBV infection and fatal EBV-associated lymphoproliferation, clinically resembling XLP. Both girls had a homozygous mutation in the gene encoding IL-2-inducible T cell kinase (Itk), a protein required for iNKT cell development in mice [56,57,58]. In line with this, one of the girls was shown to be deficient in iNKT cells (the other girl could not be tested due to lack of material) [59]. Of note, a contribution by other immune effector mechanisms cannot be ruled out, since a defect in signaling molecules such as SAP, XIAP, and Itk also affects T cell and NK cell function. 

Susceptibility of iNKT cell-deficient humans so far appears restricted to herpesviral infections. In contrast, mice lacking expression of CD1d and/or iNKT cells were found to be vulnerable to a variety of viruses, including HSV-1 [60,61], HSV-2 [62], respiratory syncytial virus (RSV) [63], and influenza virus [64]. Furthermore, activation of iNKT cells by treatment with α-GalCer protected mice in infection models studying diverse viruses, including murine cytomegalovirus (MCMV) [65], RSV [63], influenza virus [64,66], HBV [67], and diabetogenic encephalomyocarditis virus (EMCV-D) [68]. These combined observations support the view that iNKT cells contribute to anti-viral defense. Similar to mice, iNKT cell-deficient humans are probably susceptible to other types of viruses as well, which will be an interesting topic of future research. 

## 6. Viral Evasion of CD1d-Resticted Antigen Presentation

**Figure 2:**
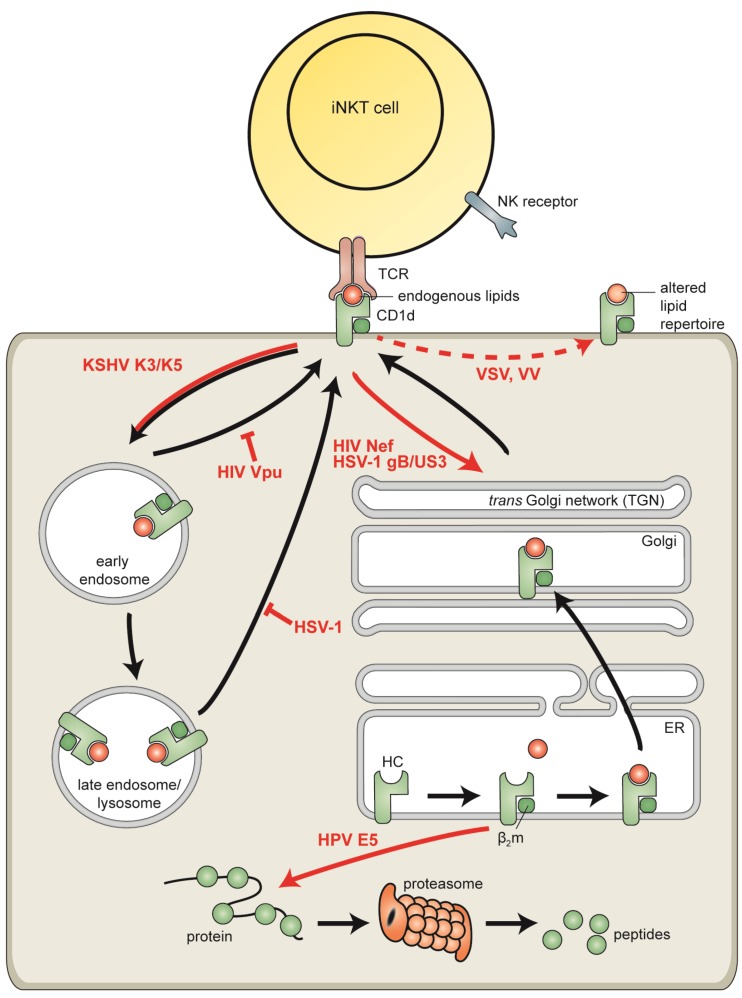
Viral evasion of CD1d-restricted lipid presentation to invariant natural killer T (iNKT) cells. Viruses subvert CD1d-restricted antigen presentation using different strategies: degradation of CD1d by cytosolic proteasomes (human papillomavirus (HPV) E5), redirection of CD1d to the *trans*-Golgi network (human immunodeficiency virus (HIV) Nef and herpes simplex virus type 1 (HSV-1) gB/US3), increased endocytosis of cell surface CD1d (Kaposi’s sarcoma-associated herpesvirus (KSHV) K3 and K5), inhibition of CD1d recycling from endolysosomal compartments to the cell surface (HIV Vpu and HSV-1), and modulation of MAPK-dependent CD1d trafficking, thereby presumably affecting the lipid repertoire presented by CD1d molecules (vesicular stomatitis virus (VSV) and vaccinia virus (VV)). HIV gp120 downregulates CD1d surface expression via an unknown mechanism.

Considering the importance of iNKT cells in anti-viral defense, it may not come as a surprise that viruses have acquired strategies to modulate detection by iNKT cells. Viruses, and herpesviruses in particular, are well known to evade cytotoxic T lymphocyte (CTL) detection by reducing MHC class I surface display [1,2,3]. More recently, the first observations on virus-induced CD1d downregulation and iNKT cell evasion were reported, as will be discussed below (Figure 2). 

### 6.1. Human Immunodeficiency Virus

Human immunodeficiency virus (HIV), the causative agent of acquired immune deficiency syndrome (AIDS), is a retrovirus that infects CD4^+^ cells (including T cells, NKT cells, macrophages and DCs). HIV infection is generally not cleared by the host immune system, resulting in chronic infection. To prevent elimination by T cells, HIV interferes with MHC class I- and class II-restricted antigen presentation. For instance, the HIV Nef protein causes downregulation of mature MHC class II complexes at the cell surface while display of immature MHC class II is increased [69]. More recently, HIV has been shown to also escape iNKT cell recognition (reviewed in [70]). A marked depletion of iNKT cells is observed after HIV infection, most likely resulting from cytolytic infection combined with activation-induced cell death. HIV further escapes iNKT cell recognition by downregulating CD1d surface display. Three viral proteins were shown to be involved in this process. Incubation of cells with recombinant HIV gp120 protein resulted in downregulation of CD1d surface levels [71], although the mechanism of action remains to be elucidated. HIV Nef accelerates the internalization of CD1d from the plasma membrane, retaining these lipid-presenting molecules in the *trans*-Golgi network [72]. Nef-induced downregulation acts via the tyrosine-based targeting motif located in the cytoplasmic tail of CD1d [72,73]. This is reminiscent of the requirement for the tyrosine residue in the cytoplasmic tail of MHC class I molecules that is involved in AP-1 binding underlying Nef-induced CTL evasion [74]. Whereas MHC class I molecules are then redirected to lysosomes [74], Nef targets CD1d molecules towards the *trans*-Golgi network [72]. Finally, HIV Vpu retains CD1d molecules in early endosomes, thereby impairing recycling of CD1d from endocytic compartments to the cell surface [75].

It is intriguing that a virus encoding few gene products like HIV codes for at least three proteins that inhibit CD1d-mediated lipid presentation, suggesting that iNKT cells are particularly important in the control of HIV infection. 

### 6.2. Vaccinia Virus and Vesicular Stomatitis Virus

Vaccinia virus (VV), the prototypic member of the family *Poxviridae*, has been successfully used as a vaccine to effectuate the eradication of smallpox. Poxviruses are large enveloped DNA viruses encoding ~200 proteins. In contrast, vesicular stomatitis virus (VSV) is a small single-stranded RNA virus belonging to the family *Rhabdoviridae* and codes for only five proteins. Interestingly, both viruses were found to interfere with CD1d-restricted lipid presentation by a similar mechanism. 

Infection with either VV or VSV resulted in reduced activation of iNKT cells, although CD1d surface levels remained unchanged. The two viruses modulated MAPK signaling and subsequent intracellular CD1d trafficking, thereby presumably altering the lipid repertoire presented by CD1d for iNKT cell recognition [76]. The VV-encoded proteins B1R and H5R were found to be involved in evasion from CD1d-restricted iNKT cells [77]. Yet, B1R is a viral kinase that phosphorylates H5R, a transcription factor involved in late viral protein expression, and thus the effects of B1R and H5R on iNKT cell evasion may be indirect. 

The use of a similar mechanism to thwart iNKT cell recognition by two unrelated viruses, like VV and VSV, could indicate this to be a general CD1d evasion strategy of viruses. However, infection with the small single-stranded RNA virus lymphocytic choriomeningitis virus (LCMV) had no direct effect on NKT cell activation [76] and thus interference with CD1d-restricted antigen presentation seems to be virus-specific. 

### 6.3. Human Papillomaviruses

Human papillomaviruses (HPV) are small DNA viruses infecting epithelia of the skin (cutaneous HPV subtypes) or mucous membranes (genital HPV subtypes). Whereas infection with low-risk cutaneous HPV types causes benign warts, the high-risk genital HPV types are associated with cervical cancer. 

HPV type 16 inhibits MHC class I- and class II-restricted peptide presentation through expression of the small hydrophobic E5 protein [78,79,80]. In addition, HPV interferes with CD1d-resticted lipid presentation. In immunostained clinical samples, CD1d molecules were abundantly expressed by uninfected cervical epithelium, but undetectable in HPV-infected lesions. Expression of HPV E5 proteins in cell lines caused a reduction in both cell surface and total CD1d protein levels. HPV E5 interacts with calnexin and prevents exit of CD1d molecules from the ER. Although mechanistic details are lacking, upon cellular expression of HPV E5, CD1d molecules end up in the cytosol, where they are degraded by the proteasome. The ability to interfere with CD1d-mediated antigen presentation is conserved among the E5 proteins of a low-risk (HPV6) and a high-risk (HPV16) subtype [81]. 

### 6.4. Kaposi’s Sarcoma-Associated Herpesvirus

Kaposi’s sarcoma-associated herpesvirus (KSHV) is the most recently discovered human gamma-herpesvirus and is implicated in the development of malignancies, such as Kaposi’s sarcoma, primary effusion lymphoma, and multicentric Castleman’s disease [82]. Herpesviruses are large enveloped DNA viruses that persist for life in infected hosts, mostly as a latent infection with limited viral gene expression. Intermittent periods of reactivation allow transmission of herpesviruses to new hosts. The herpesvirus family is famous for its large variety of immune evasion tactics, affecting many aspects of the anti-viral immune response [83,84,85,86,87]. 

During productive KSHV infection, two immunoevasins, K3 and K5 (also known as modulator of immune recognition (MIR) 1 and 2, respectively), interfere with MHC class I-restricted antigen presentation. Both K3 and K5 are membrane-bound E3-ubiquitin ligases that ubiquitinate the cytosolic tails of MHC class I heavy chains, causing their enhanced endocytosis and subsequent lysosomal degradation [88,89]. Besides MHC class I, K5 reduces surface display of the co-stimulatory molecules B7.2 and ICAM-1 [90,91].

Likely based on similarities with MHC class I, CD1d molecules are also prone to K3- and K5-induced downregulation. Although CD1d molecules enter the endocytic pathway as a consequence of K5-mediated ubiquitination of their cytoplasmic tails, the total cellular levels of CD1d remain virtually unchanged. Thus, lysosomal degradation of CD1d is not increased in cells expressing KSHV K5 [92]. The difference in this aspect between MHC class I and CD1d most likely originates from their susceptibility to lysosomal degradation, with MHC class I molecules being susceptible and CD1d/β2m complexes being resistant. Finally, K5 expression inhibited CD1d-restricted iNKT cell activation. This, combined with observations that surface CD1d levels are reduced on B cells undergoing productive KSHV infection [92], might suggest that KSHV not only thwarts CTL, but also iNKT cell activation. 

### 6.5. Herpes Simplex Virus

HSV-1 is a human alpha-herpesvirus and the causative agent of cold sores. Similar to other herpesviruses, HSV-1 hampers immune activation at multiple levels. For instance, HSV-1 inhibits MHC class I-restricted T cell recognition by combining a block in antigenic peptide import into the ER, exerted by ICP47, with a general reduction in host cell protein synthesis, imposed by the virion host shutoff (vhs) protein [93]. 

Downregulation of CD1d at the surface of HSV-1-infected cells is independent of host shutoff and total protein levels of CD1d are not reduced by HSV-1 infection. Instead, HSV-1 downregulates CD1d surface display by inhibiting recycling of CD1d molecules from endosomal compartments to the cell surface, leading to redistribution of CD1d to the limiting membranes of lysosomes. This HSV-1 induced downregulation is independent of the cytoplasmic tail of CD1d [94]. 

Two HSV-1 proteins were recently found to cooperatively hamper CD1d-mediated antigen presentation to iNKT cells, namely gB, a major viral glycoprotein, and US3, a viral serine-threonine kinase. gB was essential, but not sufficient, for CD1d downregulation during viral infection. Efficient CD1d downregulation required co-expression of an active US3 enzyme that modulates gB trafficking. In line with this, HSV-mediated inhibition of iNKT cell activation was reduced for US3-deficient viruses and abolished for gB-deficient viruses. HSV gB proteins directly bind to CD1d complexes within the ER and their association is maintained throughout intracellular trafficking. CD1d trafficking is altered by the concerted action of gB and US3, redirecting CD1d to the *trans*-Golgi network [95]. These observations seem in contrast with a previous study, reporting CD1d to be redirected to lysosomes [94]; the use of different HSV-1 strains may explain this apparent inconsistency. In addition to CD1d, HSV-1 gB is also involved in altered trafficking of MHC class II molecules [96,97]; whether similar mechanisms are underlying the manipulation of CD1d- and MHC class II-restricted antigen presentation will be an interesting topic for further research. 

Reduced CD1d display was observed on DCs infected with high levels of HSV-1, but, unexpectedly, increased surface levels of CD1d were found when DCs contained low viral loads. Despite this, DCs infected with either low or high HSV titers were both crippled in their capacity to stimulate cytokine secretion by iNKT cells [98]. Therefore, additional mechanism(s), independent of surface CD1d downregulation, are likely employed by HSV to interfere with iNKT cell function. Interestingly, a recent study by Bosnjak *et al*. showed that HSV-1 infection of keratinocytes hampered the activation of iNKT cells independent of CD1d downregulation. Instead, coculture with HSV-1 infected cells did not impair TCR triggering but rather signaling downstream of the TCR within the iNKT cells. This inactivation of iNKT cells required direct contact between the HSV-1 infected cells and the iNKT cells and was sustained after removal of the infected cells [99]. 

## 7. Concluding Remarks

In recent years, anti-viral defense mechanisms have been found to include innate immune effectors. Among these are iNKT cells, versatile cytokine-secreting cells with strongly polarizing effects. Appearance of virus-related disease in both iNKT cell-deficient patients and CD1d or Jα18 knock-out mice provides solid evidence for the importance of iNKT cell activity in the control of viral infection. 

Viral interference with CD1d-restricted antigen presentation further underscores the contribution of iNKT cells to anti-viral defense. Distinct gene products from multiple viruses (HIV, VV, VSV, HPV, and herpesviruses) have been found to act through diverse mechanisms to prevent CD1d-mediated lipid presentation to iNKT cells (Figure 2). iNKT cell interference is likely to result from the cooperative action of multiple viral gene products, analogous to T cell evasion. For instance, in cells infected with alpha- or gamma-herpesviruses, both a virus-induced block to protein synthesis and dedicated inhibitors of MHC-restricted antigen presentation contribute to downregulating surface MHC class I and/or class II display, thereby efficiently inhibiting T cell recognition [88,89,93,97,100,101,102,103,104,105,106,107,108]. iNKT cell escape appears to be effectuated by, at least, three HIV proteins and two cooperatively acting HSV-1 gene products. Interestingly, a number of the viral proteins described to reduce CD1d surface expression were already known for their ability to downregulate MHC class I or class II. Examples include (ubiquitination-induced) enhanced endocytosis of CD1d molecules from the surface of cells expressing viral proteins. We anticipate that additional iNKT cell evasion strategies will be discovered by scrutinizing other viral MHC class I or class II inhibitors for their influence on CD1d-mediated lipid presentation. Studies on CD1d viral escape mechanisms provide novel insights into pathogen-host interactions. Moreover, investigating how viral infection affects iNKT cell activation will yield further understanding of conventional CD1d-mediated lipid presentation. 

Whereas a number of bacteria-derived lipid antigens have been identified that are capable of eliciting an iNKT cell response when presented as lipid/CD1d complexes, the identity of antigenic lipids presented during viral infection remains currently unknown. Virus-specific lipids do not exist and, therefore, altered presentation of self-lipids must be responsible for iNKT cell activation during viral infection. TLR-mediated signals are likely key in driving these alterations. The identification of such virus-induced CD1d antigens poses a challenge for the coming years. 
